# De Novo Transcriptome Assembly and Population Genetic Analyses for an Endangered Chinese Endemic *Acer miaotaiense* (Aceraceae)

**DOI:** 10.3390/genes9080378

**Published:** 2018-07-27

**Authors:** Xiang Li, Meng Li, Lu Hou, Zhiyong Zhang, Xiaoming Pang, Yingyue Li

**Affiliations:** 1 National Engineering Laboratory for Tree Breeding, College of Biological Sciences and Technology, Beijing Forestry University, Beijing 100083, China; lx2016bjfu@163.com (X.L.); limeng@bjfu.edu.cn (M.L.); houlu822@163.com (L.H.); xmpang@bjfu.edu.cn (X.P.); 2 Beijing Key Laboratory of Ornamental Plants Germplasm Innovation and Molecular Breeding, National Engineering Research Center for Floriculture, Beijing Laboratory of Urban and Rural Ecological Environment, Key Laboratory of Genetics and Breeding in Forest Trees and Ornamental Plants of Ministry of Education, School of Landscape Architecture, Beijing Forestry University, Beijing 100083, China; zhangzhiyong543@163.com

**Keywords:** *Acer miaotaiense*, transcriptome sequencing, genetics diversity, population structure, conservation implication

## Abstract

*Acer miaotaiense* (P. C. Tsoong) is a rare and highly endangered plant in China. Because of the lack of genomic information and the limited number of available molecular markers, there are insufficient tools to determine the genetic diversity of this species. Here, 93,305 unigenes were obtained by multiple assembled contigs with a transcriptome sequencing program. Furthermore, 12,819 expressed sequence tag-derived simple sequence repeat (EST-SSR) markers were generated, 300 were randomly selected and synthesized, 19 primer pairs were identified as highly polymorphic (average number of alleles (N_a_) = 8, expected heterozygosity (H_e_) = 0.635, polymorphism information content (PIC) = 0.604) and were further used for population genetic analysis. All 261 samples were grouped into two genetic clusters by UPGMA, a principal component analyses and a STRUCTURE analyses. A moderate level of genetic differentiation (genetic differentiation index (F_st_) = 0.059–0.116, gene flow = 1.904–3.993) among the populations and the major genetic variance (81.01%) within populations were revealed by the AMOVA. Based on the results, scientific conservation strategies should be established using in situ and ex situ conservation strategies. The study provides useful genetic information for the protection of precious wild resources and for further research on the origin and evolution of this endangered plant and its related species.

## 1. Introduction

*Acer miaotaiense* (P. C. Tsoong), a perennial woody plant belonging to Acer (family Aceraceae), is an endangered species mainly distributed in the Gansu and Shaanxi Provinces of China [1,2]. *A. miaotaiense* was listed as a third-class nationally protected plant in the Chinese Red Data Book in 1992 [3] and was considered a vulnerable species in the International Union for the Conservation of Nature in 2004 [4]. Because of over-exploitation, the growth and breeding of *A. miaotaiense* are seriously threatened, and the outlook is not good. As a distinctive species among the Aceraceae family, *A. miaotaiense* has nearly horizontal fruit wings and, therefore, is of great significance in studies of the morphological evolution and classification of Aceraceae. Additionally, it is a popular landscaping tree [2], and its bark, leaves and fruit can be used as raw materials for tannin extraction, resulting in *A. miaotaiense* having a large economic and ornamental value. However, a high seed abortion rate, weak natural regeneration, strict habitat conditions and frequent human exploitation have significantly reduced the number of natural populations and narrowed the geographic distribution range of *A. miaotaiense*, which resulted in it being classified as an endangered plant [5].

Studies regarding *A. miaotaiense* were mainly concerned with raising seedlings, fruiting characteristics and reproductive biology [6,7,8], and only a few genetic studies focused on population genetics and protection biology [9,10]. To protect and utilize precious wild resources of endangered species, it is necessary to have adequate molecular techniques to evaluate germplasm and study population and conservation genetics. Molecular markers have been widely used in genetic diversity analyses, marker-assisted selection, genetic map construction and evolutionary studies in plants and animals [11,12,13,14]. Compared with other traditional DNA-based molecular markers, microsatellites simple sequence repeats (SSRs) are effective markers for studying population genetics because of their high information content, codominance and high polymorphism rate [15,16]. Currently, a large number of studies, including those on genetic diversity, population structure and protective biology, have used SSRs isolated based on next-generation sequencing in endangered plant species, such as *Origanum compactum* [17], *Phellodendron amurense* [18] and *Centaurea borjae* [19].

Because of a lack of available genomic information, it was difficult to develop ideal SSR markers and to evaluate the genetic diversity and evolution of endangered species at the genome level [20,21,22]. Transcriptome sequencing represents an alternative to whole-genome sequencing because it is more cost-effective and can target genomic regions for important traits through corresponding expressed sequence tag (EST) sequences, which can be selected to develop abundant EST-SSRs [23,24]. These transcriptome sequencing-based markers not only have the characteristics of a high polymorphism rate and codominance, but also directly reflect expression, which was determined to be relatively conservative [25]. Because on these advantages, EST-SSRs have been widely used in plants, including *Pinus dabeshanensis* [26], *Ziziphus jujube* [27], *Paeonia suffruticosa* [28] and *Robinia pseudoacacia* [29], to construct genetic linkage maps, study genetic diversity and population structure, and construct core collections [30]. Therefore, transcriptome sequencing is a more forceful method for developing microsatellite markers than whole-genome sequencing. However, only a circumscribed number of SSRs were verified in Aceraceae [31,32]. To comprehensively analyze the genetic diversity and population structure of *A. miaotaiense* in natural populations in China, developing SSR markers by transcriptome sequencing is a first step. This will aid in providing genetic protection for natural resources.

In this study, for the first time, we developed EST-SSRs based on transcriptome sequencing data. These were utilized in studying genetic diversity and population structure and in further implementing feasible production strategies for this rare and vulnerable species, *A. miaotaiense*. Here, 93,305 unigenes with an average of 668 base pairs (bp) and an N50 value of 1138 bp were identified. A total of 19 EST-SSRs with high polymorphism rates were selected to assess the genetic diversity among 261 *A. miaotaiense* individuals and the structures of their six natural populations in the Qinling Mountain areas of China. The diversity analyses and EST-SSR markers described in this study will contribute to the further studies on the protection and utilization of germplasm resources of *A. miaotaiense*.

## 2. Materials and Methods

### 2.1. Plant Materials

**Figure 1:**
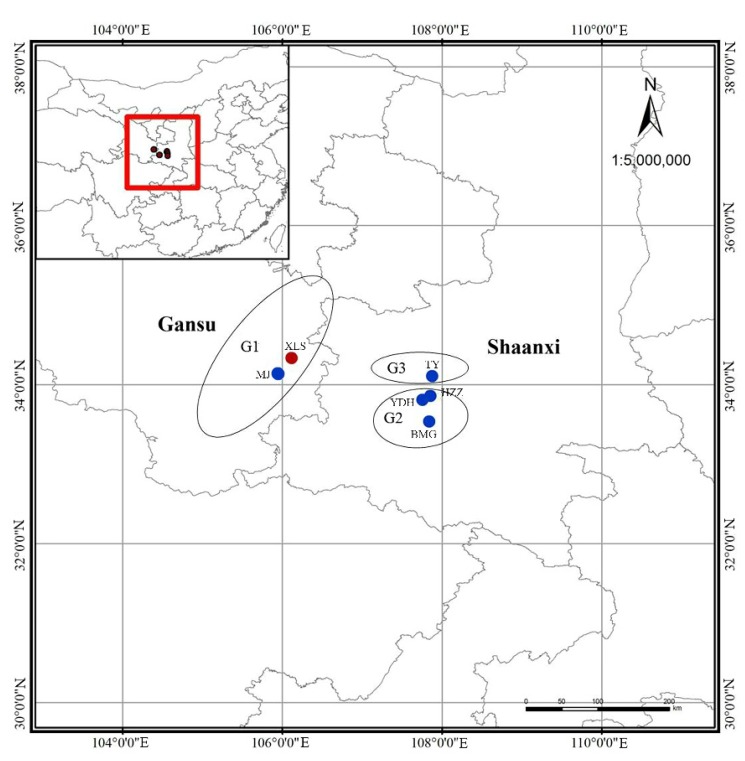
Geographic distribution of *Acer miaotaiense* collected in Qinling Mountains, China. Red dots represent genetic cluster C1, blue dots represent genetic cluster C2.

Based on our field investigation in August 2017, a total of 261 *A. miaotaiense* individuals representing six wild populations were collected in this study, which contains its main distribution regions in China (Table 1 and Figure 1). To select representative samples, all collected individuals from six populations were spaced at least 50 m apart. All of six populations were identified from five sampling locations from two different regions, including Shaanxi province and Gansu province in China. Population Xiaolongshan (XLS) and Maiji (MJ) contained samples from Gansu, China. The samples from Shaanxi province composed by three locations, which included four populations: Houzhenzi (HZZ), Yuduhe (YDH), Baimagou (BMG) and Tangyu (TY). The altitude of all populations ranged from 1260 m (BMG) to 1520 m (MJ), and the average altitude was 1430 m (Table 1). Depending on geographical location, these sampled populations were further divided into three groups (G1, G2 and G3), respectively. The West of Qinling Mountains (G1) group consisted of XLS and MJ. North of the midland of the Qinling Mountains group (G3) contained TY. South of the midland of the Qinling Mountains group (G2) included HZZ, YDH and BMG (Figure 1). At the same time, the important phenotype characteristics (leaf length, leaf width and fruit shape) of all individuals was recorded.

### 2.2. RNA Extraction, Library Preparation, Sequencing and De Novo Assembly

Three fresh tissues (leaves, fruits and phloem) were frozen and stored instantly in liquid nitrogen prior to RNA extraction. Total RNA was isolated from all the tissues collected above using the method described by Ghawana et al. [33] and was detected using NanoDrop 2000 (Thermo Fisher Scientific, Wilmington, DE, USA) and the RNA Nano 6000 Assay Kit of the Agilent Bioanalyzer 2100 system (Agilent Technologies, Palo Alto, CA, USA). The RNA of three tissues are mixed to build sequencing libraries with three biological duplications. Sequencing libraries were constructed using NEBNext^®^Ultra™ RNA Library Prep Kit for Illumina^®^ (New England Biolabs, Ipswich, MA, USA). Briefly, purified mRNA was obtained from total RNA (1 µg) per sample using poly-T oligo-attached magnetic beads; then, the mRNA was fragmented using divalent cations under elevated temperature in NEBNext First Strand Synthesis Reaction Buffer (5X). First, strand cDNA was produced based on the random hexamer primer and M-MuLV Reverse Transcriptase. Immediately after, a second strand cDNA was synthesized using DNA polymerase I and RNase H. After adenylation reaction of 3′ ends of DNA fragments, NEBNext Adaptor containing hairpin loop structure were ligated for hybridization. The fragments in library were purified with AMPure XP system (Beckman Coulter, Danvers, MA, USA) to firstly select for 240 bp cDNA fragments; 3 μL USER Enzyme (New England Biolabs) was incubated with size-selected, adaptor-ligated cDNA for 15 min at 37 °C and then 5 min at 95 °C. Then PCR reaction was performed using Phusion High-Fidelity DNA polymerase, Universal PCR primers and Index (X) Primer. Finally, PCR products were purified by AMPure XP system and the Agilent Bioanalyzer 2100 system was used to assess sequencing library quality and the clusters were sequenced on an Illumina Hiseq 2000 platform and paired-end reads were generated. Sequenced raw data described in this study were uploaded in the Sequence Read Archive (SRA) public database of the NCBI (No. SRR7195583).

Before assembly, the raw paired-end reads were firstly used to obtain high-quality clean reads. In addition, the Q20, Q30, GC-content and sequence duplication level of the clean data were calculated. Afterwards, transcriptome assembly was implemented using Trinity [34] and the min_kmer_cov was set to 2 (other parameters were set as default). The RNA library preparation and de novo assembly and further deep analyses were performed by Biomarker Technologies Co., Ltd. (Beijing, China).

### 2.3. Sequence Annotation and Classification 

For functional annotation of the assembled unigenes, these sequences were aligned to the public databases by BLASTX program (*E*-value < 1.00 × 10^−5^), including Nr (NCBI non-redundant protein sequences database); Pfam (Protein family database); KOG (eukaryotic orthologous groups); COG (Clusters of Orthologous Groups); Swiss-Prot (Swiss Institute of Bioinformatics databases); eggNOG (Evolutionary genealogy of genes: Non-supervised Orthologous Groups); KEGG (Kyoto Encyclopedia of Genes and Genomes databases); The representative GO (Gene Ontology) annotation was performed by the software Blast2GO [35].

### 2.4. Simple Sequence Repeat Loci Identification and Primer Design

The transcriptomes were analyzed using the MIcroSAtellite identification tool (MISA) (http://pgrc.ipk-gatersleben.de/misa/misa.html) for identifying SSRs with a minimum of ten repeats for di-nucleotide motifs, five repeats for tri- and tetra-, four repeats for penta- and hexa-nucleotide motifs. The primer for each SSR locus was designed using Primer3 version 0.4.0 (http://primer3.sourceforge.net/releases.php). The criteria for designing primers were as follows: primer length of 18–24 nucleotides; GC content of 40–60%; annealing temperature between 55 and 60 °C with 55 °C as optimum; PCR product size range of 100 to 350 bp.

### 2.5. DNA Isolation, PCR Amplification and Simple Sequence Repeats Validation

Total genomic DNA was extracted from 261 individuals from silica-dried leaves using the Plant Genomic DNA Kit (TianGen Biotech, Beijing, China) and was used to check DNA integrity and purity using 1% agarose gel electrophoresis and NanoDrop 2000 (Thermo Fisher Scientific). PCR amplification of each locus was implemented as shown by [36]. In addition, 300 primer pair sequences containing SSRs were randomly selected and were successfully screened in 16 individuals randomly selected among six populations for development and assessment of the polymorphism EST-SSR. Primers were synthesized by Sangon Biotech (Shanghai, China). To validate the SSR locus, the universal M13 sequence labeled by four fluorescent dyes (ROX, HEX, FAM and TAMRA) was added at the 5′ end of all forward primers.

### 2.6. Statistical Analyses

GeneMarker (version 1.5) [37] was used to obtain and analyze the microsatellite raw data obtained by the high performance capillary electrophoresis (HPCE). The genetic parameters of polymorphic loci were calculated by the GenAlEx software (version 6.5) [38], including observed heterozygosity (H_o_), H_e_, N_a_, effective number of alleles (N_e_), F_st_, inbreeding coefficient (F), Hardy–Weinberg equilibrium (HWE) and number of rare alleles (NRA). The gene flow (N_m_) was calculated as N_m_ = (1 − F_st_)/4 × F_st_ based on the F_st_ values. The PIC values of each SSR primer were obtained using the PIC calculation (PICcalc) progress [39]. To obtain the genetic variation of populations—including within populations, among populations, within groups and among groups—analyses of molecular variance (AMOVA) function of GeneAlEx version 6.5 was performed.

The Bayesian clustering analysis was implemented to evaluate the population genetic structure of *A. miaotaiense* populations using STRUCTURE version 2.3 [40]. Models were tested for K-values (testing from K = 1 to K = 10), and each independent run was performed by a burn-in period of 100,000 iterations and 100,000 Markov chain Monte Carlo repetitions. The K for the number of populations was assessed using the delta-K method by Structure Harvester program [41]. In addition, Nei’s genetic distance [42] was calculated and used to construct an unweighted pair-group method with arithmetic averaging (UPGMA) phylogenetic tree using the PowerMarker program [43]. In order to obtain more intuitionistic genetic classification, interactive Tree Of Life (iTOL) [44] was further employed to make the display, manipulation and annotation of phylogenetic trees. The principal component analyses (PCA) was calculated and performed using the GenAlEx version 6.5 program.

Based on the longitude and latitude recorded by a global positioning system (GPS), the geographic distribution of the six populations was identified by the ArcGIS version 10.2 software (ESRI, Redlands, CA, USA). The geographic distance among the sampled locations was calculated by the Geographic Distance Matrix Generator (GDMG) version 1.2.3 software (http://biodiversityinformatics.amnh.org/opensource/gdmg/index.php) (AMNH, New York, NY, USA). The relative migration networks among the six populations was performed using the function ‘divMigrates’ [45] from ‘diveRsity’ package [46] in R (version 3.5.0) [47].

## 3. Results

### 3.1. Sequencing, De Novo Assembly and Functional Annotation of Unigenes

To comprehensively overview the transcriptome information, total RNA samples from three different tissues of an adult *A. miaotaiense* tree were used to sequence and generate a de novo assembly. A total of 21,812,509 raw sequencing reads were generated on the Illumina sequencing platform (Table S1). After data filtering to remove low-quality sequences, 21,485,322 high-quality clean reads were obtained with an 88.82% Q30 and a 44.46% GC content. Using Trinity software, the assembly of these high-quality reads generated 93,305 unigenes with paired-end reads, and the total length of the unigenes was 62,398,390 bp, with an average length of 668 bp and an N50 value of 1138 bp (Table S2).

**Figure 2:**
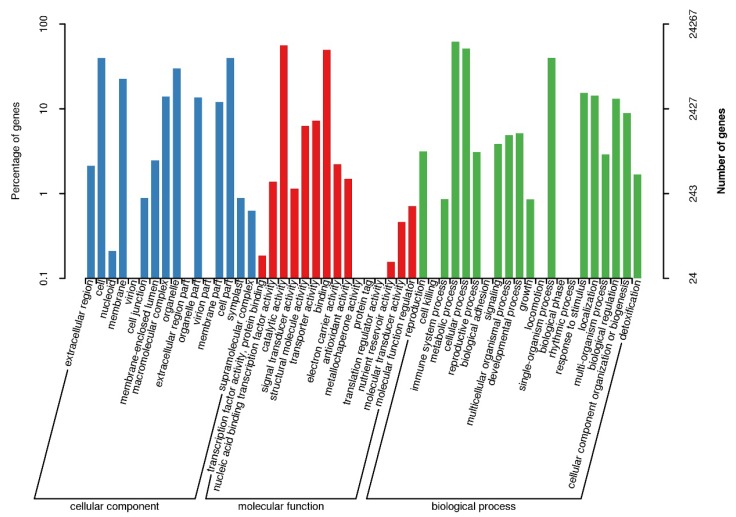
Functional classification of the assembled unigenes of *A. miaotaiense* based on gene ontology (GO) categories.

All of the *A. miaotaiense* unigenes were annotated into eight public databases. For the gene ontology (GO) functional classification, 24,267 unigenes (~45.32% of the assembled unigenes) were assigned, and these terms could be divided into three categories: molecular function (15,687, 64.65%); biological process (4580, 18.87%); and cellular component (4000, 16.48%) (Figure 2). Moreover, 27,887 unigenes (52.08%) were assigned KOG functional annotations and grouped into 26 functional categories (Figure S1). Among these categories, the group “General function prediction only” (5558, 19.93%) was dominant, followed by “posttranslational modification, protein turnover, chaperones” (2521, 9.04%) and “translation, ribosomal structure and biogenesis” (2510, 9.00%). Among the 93,305 *A. miaotaiense* unigenes, 52,023 (55.76%) were annotated to known proteins in the Nr database, with *Citrus sinensis* having the top known hits (9496, 18.25%), while unknown species represented 53.36% (27,757) (Figure S2). The analyses of the KEGG pathways based on assembled unigenes revealed that 19,473 (20.87%) were annotated and contained 131 biological pathways. Of these pathways, the ribosome (ko03010, 1677 unigenes, 7.89%) had the highest number of unigenes among the genetic information processing categories, followed by carbon metabolism (ko01200, 1185, 5.58%) and biosynthesis of amino acids (ko01230, 858, 4.04%) among the metabolism categories (Table S3).

### 3.2. Frequency and Distribution of Simple Sequence Repeats

Table S4 presents the 12,765 potential EST-SSRs that were identified in 8655 (9.28%) unigenes, of which 3049 sequences contained more than one EST-SSR locus and 1587 SSRs were present in compound formation. The average microsatellite density of the *A. miaotaiense* transcriptome was one per 4888 bp. The mono-nucleotide was the most common type of repeat (5814, 52.01%), followed by di-nucleotide (2763, 24.72%), tri-nucleotide (2362, 21.13%), tetra-nucleotide (170, 1.52%), penta-nucleotide (45, 0.40%) and hexa-nucleotide (24, 0.21%) repeats. In addition, the lowest number of repeat motifs per locus was five, with 10 tandem repeats of the EST-SSR (1980, 17.71%) being the most common. This was followed by six (1310, 11.72%), 5 (1297, 11.60%) and 11 (1165, 10.42%) repeats. The other numbers of the tandem repeats each accounted for <10% of the EST-SSRs (Table S5). The most dominant type was A/T (5803; 51.91%), followed by AG/CT (836, 7.48%), GA/TC (689, 6.16%), AT/AT (619, 5.54%), TA/TA (431, 3.86%), GAA/TTC (238, 2.13%), AGA/TCT (201, 1.80%) and AAG/CTT (158, 1.41%). Thus, the AG/CT and GAA/TTC repeats were the most abundant di- and tri-nucleotide motifs, respectively, in *A. miaotaiense* (Table S6).

### 3.3. Development and Polymorphism Rates of New Expressed Sequence Tag-Derived Simple Sequence Repeats Markers

In this study, 300 primer pair sequences containing SSRs were randomly selected from 1213 primer pairs designed by Primer3. We removed mono-nucleotide repeats from our analyses. Of the 300 selected primer pairs, 96 successfully PCR-amplified *A. miaotaiense* genomic DNA (Table S7), producing products of the expected size. The remaining primer pairs failed to generate the expected PCR products. All 96 successful primer pairs were used in 16 *A. miaotaiense* individuals to validate the polymorphisms by capillary electrophoresis. Of these, 19 primer pairs were highly polymorphic, and the remaining primer pairs were identified as monomorphic (Table 2). In total, 152 alleles were detected across the collected individuals, with a mean value of eight observed alleles per locus. The number of alleles varied from three (BFAM-107 and BFAM-254)) to 19 (BFAM-166). Among the 19 microsatellites, 35 of 152 rare alleles were found at 19 loci, with an average of 1842. In addition, the PIC values for all of the loci ranged from 0.147 (BFAM-254) to 0.820 (BFAM-219), with an average of 0.604, and 14 of 19 loci had highly informative scores (PIC > 0.50) (Table 2).

### 3.4. Genetic Diversity and Differentiation

The genetic diversity levels of the six *A. miaotaiense* populations were estimated in Table 3. The highest level of genetic diversity occurred in XLS (N_a_ = 6.053, N_e_ = 2.965, H_o_ = 0.512 and H_e_ = 0.573), while the lowest diversity levels were in BMG (N_e_ = 2.561, H_o_ = 0.464 and H_e_ = 0.504) and TY (N_e_ = 3.173, H_o_ = 0.496 and H_e_ = 0.585). The fixation index (F) values of the six populations varied from −0.046 to 0.147, with a positive average, which indicated that the heterozygote is deficient, the homozygote is excessive and significant inbreeding occurred in the *A. miaotaiense* populations (Table 3).

**Figure 3:**
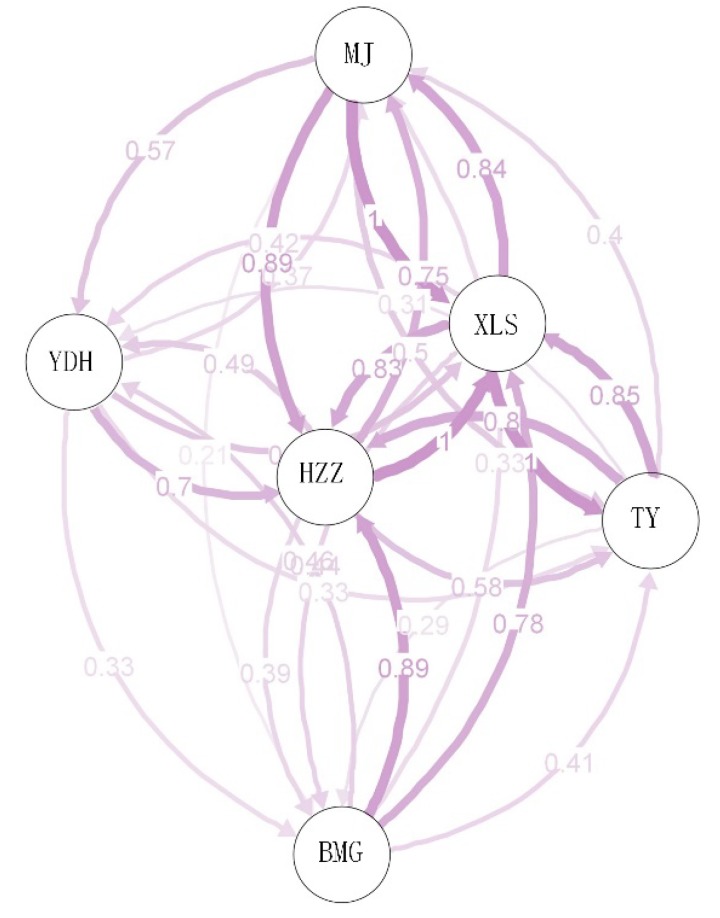
The relative migration networks of six populations from divMigrates. The width of the line and the number shown next to the arrows indicate the migration rate.

Genetic distances and pairwise F_st_ values calculated between populations are listed in Table 4, and they ranged from 0.173 to 0.401 and from 0.059 to 0.116, respectively. The highest genetic distance value (0.401) was observed between MJ and BMG populations, while the lowest genetic distance value (0.173) was observed between XLS and TY populations. The pairwise F_st_ values and the genetic distances corresponded. The greatest level of differentiation occurred between MJ and BMG populations, while the lowest level of differentiation occurred between MJ and HZZ populations (Table 4). In this study, the AMOVA analyses revealed 81.01% genetic variance within populations, which indicated a high genetic diversity level within populations (Table 5). However, the variance component among populations (18.99%) was lower than that within populations. In groups divided based on their geographical distribution, a lower genetic differentiation level (1.44%) among the three groups was detected by AMOVA (Table 5). The relative migration networks among the six populations were estimated by divMigrate (Figure 3). A high level of gene flow was observed in the XLS population (from Gansu Province), while the other five populations grouped together exhibited a relatively low gene flow. The lowest gene flow value was 1.905, which was observed between the MJ and BMG populations, while the highest was 3.987, which was observed between the MJ and HZZ populations (Table S8).

The geographic distances for all six wild populations were calculated using latitude and longitude, and ranged from 35.8 km to 190.6 km. Genetic distance is estimated by pairwise F_st_/(1 − F_st_) among different populations, and then is regressed against the geographic distance. Mantel’s test indicated that the correlations established using the reduced major axis regression for genetic distance value and the geographic distance were very weak among the five populations (*r^2^* = 0.35, *p* = 0.50). The greatest geographic distance was found between MJ and BMG, and the Rousset’s distance between them was also the largest. Of the Rousset’s distances, that between MJ and HZZ was the lowest. Thus, there was no significant correlation between genetic and geographic distances, which indicated that the geographic distances observed among populations were not key factors influencing genetic differentiation (Figure S3).

### 3.5. Population Structure of A. miaotaiense

**Figure 4:**
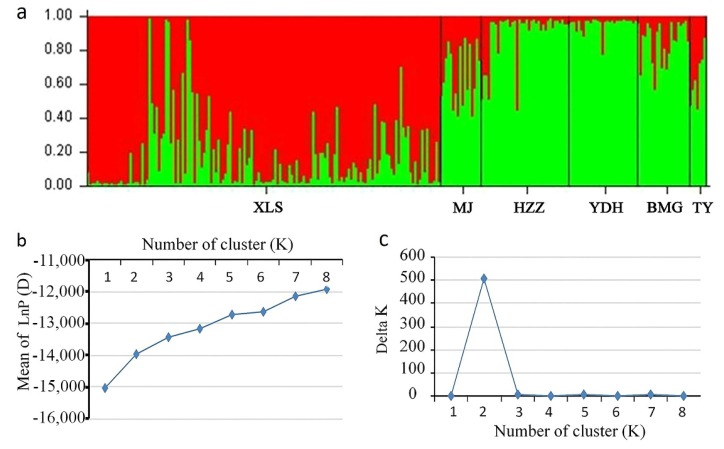
Population structure of 261 *A. miaotaiense* individuals based on 19 EST-SSRs markers. (**a**) Estimated genetic structure of the six populations based on STRUCTURE analysis; (**b**) Estimation of population using the mean of Ln P(D) based on ten repetitions for each K value; (**c**) plot of delta K (ΔK) for K ranging from 1 to 8.

An analysis of the structures of the six populations containing 261 individuals was performed with a Bayesian-based approach using the software STRUCTURE 2.3.4. The optimal number of clusters (K) was identified based on the LnP(D) and posterior probability (ΔK) values. K was tested from one to eight with 10 repetitions performed for each run. In this study, a clear peak in the value of ΔK was obtained at K = 2 (Figure 4b,c). The tested *A. miaotaiense* individuals were grouped into two different clusters, C1 and C2 when K = 2 (Figure 1 and Figure 4a). C1 consisted of 149 individuals of one populations from the region G1 (XLS), C2 contained the remaining 112 individuals of five populations from the regions G2 (TY) and G3 (MJ, HZZ, YDH and BMG). In addition, 149 of 166 individuals collected from Gansu province were assigned into C1, whereas the remaining 17 samples grouped into C2. The admixture of the five populations (MJ, HZZ, YDH, BMG and TY) formed C2, and one population (XLS) formed the other cluster.

**Figure 5:**
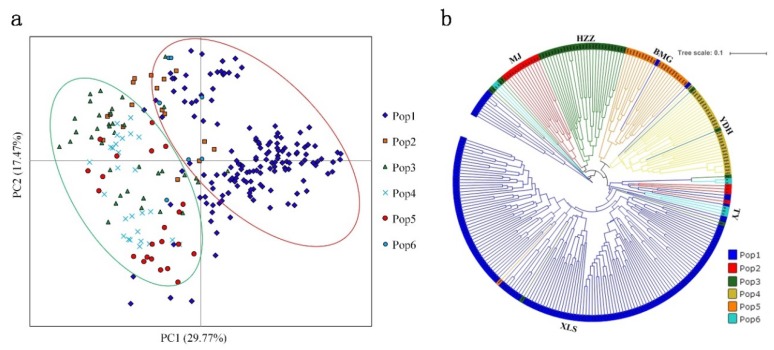
Graphical representation of differentiation between populations. (**a**) Principal component analyses (PCA). (**b**) UPGMA tree of 261 *A. miaotaiense* individuals.

To further assess the populations’ genetic structures, a PCA based on the 19 EST-SSR markers was used to generate a scatter plot of the six populations. The 261 *A. miaotaiense* individuals were clearly separated into two broad groups across the first two axes (Figure 5a). The first two principal coordinates explained 29.77% and 18.47%, individually, and a combined 48.24% of the total variation, in which the first principal coordinate separated the XLS population. However, the MJ, HZZ, YDH, BMG and TY populations were mixed together, which further supported the STRUCTURE results, because MJ clustered with HZZ, YDH, BMG and TY. In addition, *A. miaotaiense* individuals originating in G2 and G3 were clustered in C2. Furthermore, a UPGMA tree of 261 individuals was constructed based on Nei’s genetic distance (Figure 5b). Two major clusters clearly formed, and few individuals occurred as admixtures in different clusters. Populations containing 149, 17, 37, 29, 22 and 7 individuals are shown in blue, red, and green, yellow, light salmon and turquoise, respectively. These clustering results were consistent with the results of the PCA and model-based population structure analyses of the 261 *A. miaotaiense* individuals.

## 4. Discussion

Genetic diversity is the result of the long-term evolution of species or populations [48,49] and makes it possible to adapt to a changing environment. In particular, it is important to explore the causes and processes of rare or endangered species [50]. To design viable protection strategies for threatened species, a core requirement is the knowledge of genetic diversity and population structure. However, only limited publications reported on the development of molecular markers for the genetic analyses of *A. miaotaiense* [5]. Our study is the first attempt to obtain comprehensive transcriptional information for the development of EST-SSR markers and to further examine the diversity and genetic structure of extant natural *A. miaotaiense* populations.

In total, 21,485,322 high-quality clean reads were generated with 88.82% Q30 and a 44.46% GC content using Illumina paired-end sequencing, which was consistent with the results from transcriptome sequencing in *P. dabeshanensis* (44.58%) [26] and *Picrorhiza kurrooa* (44.6%) [51] but was higher than genus *Gossypiumis* (37%) [52], *Acer palmatum* (44.33%) [53] and *Chlorophytum borivilianum* (44%) [54], indicating a better quality of sequencing. Furthermore, 93,305 unigenes were obtained from the sequence assembly, with an average length of 668 bp and an N50 value of 1138 bp, which was longer than those reported—*A. palmatum* (average length = 564.78 bp, N50 = 738 bp) [52] and *Sepia esculenta* (average length = 621 bp, N50 = 911 bp) [55]—using the same technology, which might be attributable to the sequencing depth, assembly method and nature characteristics of the species. Thus, the transcriptome sequencing data of *A. miaotaiense* appeared to be of high quality and thus was utilized for further studies on marker development, population structure and genetic diversity. To understand the biological functions of assembled transcripts in *A. miaotaiense*, the functional annotations of the assembled unigenes were obtained using diverse public databases. For the GO analyses, 24,267 (45.32%) unigenes were annotated into 41 GO terms and ‘the cell part and cell’, ‘binding and catalytic activity’ and ‘cellular process and metabolic process’ were the most abundant terms in the cellular component, molecular function and biological process categories, respectively, which was consistent with GO functional categories observed in *Dipteronia oliver* [31]. In addition, 19,473 (20.87%) unigenes predicted by KEGG pathways were mapped to 131 biological pathways, and the majority of the categories were related to metabolism. The carbon metabolism (ko01200, 1185, 5.58%) and biosynthesis of amino acids (ko01230, 858, 4.04%) pathways were identified in this study. These results not only revealed the active metabolic processes in *A. miaotaiense* but also mean that multifarious metabolites are synthesized in this species. For *A. miaotaiense* and its related species, the leaves are the most important metabolic organ and contain large amounts of terpenoids, esters and aldehydes, which aid in resisting environmental and biological stresses, such as freezing, drought and pests. Moreover, some of unigenes were observed in plant hormone signal transduction (ko04075, 294, 1.38%) that responds to environmental information, such as abscisic, jasmonic and salicylic acids, which are related to stress. These results provide valuable functional annotation information for future investigations of gene function, stress responses and biological pathways in *A. miaotaiense*.

SSR markers have abundant types and are widely distributed in plant genomes [56]. In the present study, 12,765 potential EST-SSR molecular markers were identified from 93,305 unigenes, including 3049 unigenes that contained more than one EST-SSR locus, using RNA sequencing. The di-nucleotide repeats in EST-SSRs were the most abundant type in *Michelia coriacea* (Magnoliaceae) [57], *Brassica oleracea* [58], garlic [59] and *Z. jujube* [60]. In the identified 12,765 SSR markers of *A. miaotaiense*, the major repeat type was di-nucleotide (2763, 24.72%) after the removal of the mono-nucleotide repeats, which was consistent with previous findings, followed by tri-nucleotide repeats (2362, 21.13%). The most dominant di-nucleotide repeat motif of *A. miaotaiense* was AG/CT (836, 7.48%), which was consistent with studies in peanut [61] and *D. oliver* [31], but the most abundant tri-nucleotide repeat motif was different from those of other plants (GAA/TTC for *A. miaotaiense*, AAG/CTT for *D. oliver* and AAG/CTT for peanut). In particular, the CG/CG motif was not observed in *A. miaotaiense*, which further supports the conclusion that the CG/CG repeat is rare in many dicotyledonous plants [62,63,64].

Genetic diversity plays a significant role in the genetic improvement of germplasm resources, which has been widely applied in many plants [65,66,67]. The level of genetic diversity in a species is often related to the numbers of loci and populations [68], the size of the geographical range [69] and genetic exchange [70]. In the present study, there was still a high level of genetic diversity (N_a_ = 8, H_o_ = 0.528, H_e_ = 0.635 and PIC = 0.604) in wild *A. miaotaiense*. These results are similar to those determined of seven wild populations of *Acer davidii* (N_a_ = 7.182, H_o_ = 0.181 and H_e_ = 0.389) as determined by using 11 primer pairs to evaluate the genetic diversity of SSR markers, He et al., 2017 [32]. However, a low level of genetic diversity has been found for the same family in *D. oliver* (N_a_ = 4.5, H_o_ = 0.119 and H_e_ = 0.714) [31] at the species level. The main reason for this difference is that only 44 individuals collected from six natural populations were used to analyze the genetic diversity in Zhou’s study, whereas we collected 261 samples from six wild populations within the distribution range. Generally, species with small population sizes tend to maintain a low level of genetic diversity compared with widely spread species. However, there are contradictory conclusions [71]. In our study, *A. miaotaiense* is only naturally distributed in the Qinling Mountains, but its genetic diversity is higher than those of *Oryza sativa* (N_a_ = 4.8, H_o_ = 0.07 and H_e_ = 0.55) [72] and *Phaseolus vulgaris* (N_a_ = 2.7, H_o_ = 0.626 and H_e_ = 0.308) [73]. For other endangered species, such as *M. coriacea* (Magnoliaceae) [59], a restricted and fragmented distribution were determined in southeastern Yunnan Province, China, but a high level of genetic diversity and a low level of genetic differentiation were determined (N_a_ = 4.091, H_e_ = 0.505 and H_o_ = 0.412 for the SSR markers). Low levels of genetic diversity would not be beneficial in an expanded distribution region and could increase the possible susceptibility to diseases or insect pests. Thus, further studies on genetic protection should be used to construct a library of genetic diversity to preserve genetic variation within species. The distribution patterns of genetic structure are closely related to the breeding mechanisms and natural selection acting on species [74]. In this study, based on the different approaches (STRUCTURE, PCA and UPGMA), the 261 *A. miaotaiense* individuals were divided into two major distinct groups for the highest ΔK value. However, each group was more or less mixed with the lineages of the other group, which was also reported in garlic [59].

Environmental conditions and species characteristics are also considered as critical factors and may affect genetic diversity. These have been shown in other plant species, such as *Salix psammophila* [66], *Rhododendron jinggangshanicum* [75] and *Ranunculus acris* [76]. In our field investigation, many abundant morphological variations were found firstly in *A. miaotaiense*, including shoulder-shaped leaves and flat fruit with three wings. Aditionally, it is perennial and wind-pollinated, which may be one cause of the high level of genetic diversity, the number of alleles, and the high PIC value among these samples. The genetic diversity varied in *A. miaotaiense* from different populations in China. XLS had the highest level of genetic diversity, while BMG had the lowest level of genetic diversity. The XLS is located in the western edge of the distribution area whereas BMG is located 1260 m at the southern edge of the Qinling Mountains. The marginal distribution may change allele frequency and generate a low level of genetic diversity. *A. miaotaiense* is mainly distributed in the Qinling Mountains (400–500 km from east to west, with an average altitude of more than 2000 m) and is often used as an ornamental tree. A natural gene flow for *A. miaotaiense* over such a distance and altitude would be difficult, and one putative explanation may be frequent human actions. *A. miaotaiense* from different regions are often planted together, inducing gene flow and increasing diversity.

F_st_ is an effective measure of genetic differentiation and gene flow between populations [77]. Moderate population differentiation (pairwise F_st_ = 0.059–0.116) and weak population structure were found among the six natural populations. The greatest level of genetic differentiation was found between populations 2 and 5, and the distance between them was ~187 km, which indicated that long-term isolation may limit the level of gene flow between two populations. The same result was also shown in the relative migration networks of the six sampled populations. An AMOVA indicated that the greatest level of genetic differentiation in *A. miaotaiense* exists within populations (81.01%), while less genetic differentiation occurs among the populations (1.44%), and the same results have been reported in previous studies based on SSR markers [78,79]. It produced a high F_st_ value (0.19 > 0.15), indicating great differentiation among populations [80]. This may be attributed to flower heterogeneity of Aceraceae species, smooth light pollen and anemophilous pollination with an outcrossing breeding system which could improve diversity within populations while reducing genetic diversity among the populations. The woody species with predominately outcrossing tend to have less differentiation among populations and high differentiation within populations [81]. The higher value of the within-population variance in this study is likely due to little gene flow between collection sites and specific mating systems from which the samples were derived.

Genetic differentiation into different populations is strongly influenced by gene flow, long-term evolution, genetic drift, selection and mutations [82]. In this study, the genetic differentiation within three geographical groups accounted for about 80.73% of the total, suggesting that there is less differentiation among them. A relatively high level of genetic diversity was found in the XLS population in *A. miaotaiense*, which inhabits relatively independent areas of Tianshui, Gansu Province, with relatively high number of Na and private alleles. Furthermore, the F_st_ value between XLS and other populations varied from 0.065–0.089, indicating moderate levels of genetic differentiation. This variation difference may be explained by the larger sample size, adult trees and specific mating systems of XLS compared with other populations. The lowest F_st_ value was detected in MJ and HZZ populations, suggesting a low degree of genetic differentiation between them, and this finding was consistent with the UPGMA analyses. It may be that the frequent human activity and geological history of the Qinling Mountains restricted the continuity of their distribution, but that the later development of a similar environment along ditch edges guided the evolution of the two populations in the same direction. The Nm value among *A. miaotaiense* populations ranged from 1.905 to 3.987, indicating the frequent flow of genes of populations. The clustering of *A. miaotaiense* populations showed a clear separation according to the dendrogram from Nei’s genetic distance matrix by UPGMA. In terms of population structure, the populations from G2 and G3 were nearly clustered into C2, while the populations from G1 were clustered into C1, except MJ, which is not consist with the known geographical location information (G1, G2 and G3). Similar results were also found using Mantel’s test for the correlation between genetic distance value and the geographic distance matrix (*r^2^* = 0.35, *p* = 0.50). These results showed that the genetic distance was not significantly correlated with the geographic distance, suggesting that geographic distance is not the principal factor influencing genetic differentiation in *A. miaotaiense*.

Maintenance of high genetic diversity and a reasonable population structure are the core goals for the conservation of the endangered species and are of great significance in the establishment of conservation policies and scientific measures. Only few adult fruiting trees were observed in the sampled regions, among which XLS populations had a relatively greater number of adult trees with only 46 individuals. Natural regeneration is difficult and there are very few young seedlings in the forest. Thus, an effective policy should first protect the habitat and natural populations, especially populations with higher levels of genetic diversity (XLS and HZZ), and take some measures for in situ conservation, such as the establishment of natural protective areas to shelter as many individuals as possible. The greatest level of genetic differentiation was found between the MJ and BMG populations. To protect independent population units, these populations are the most important for in situ conservation to maintain as much genetic diversity as possible. Furthermore, overexploitation may be the main cause for the reduction in population sizes according to the field survey. Therefore, necessary protective policies, laws and regulations must be instituted by the government to forbid the unlawful felling and exploitation of this endangered species’ environment. To avoid further losses in population size, all of these extant populations (XLS, MJ, HZZ, YDH, BMG and TY) should be protected.

In addition, impactful ex situ conservation approaches are of great importance for maintaining *A. miaotaiense*’ natural genetic resources. Because of the current habitat loss and low seed-setting rate, the TY population only contains seven individuals, including two adult trees, and its genetic diversity level was the lowest. The principal protection of the TY population should involve ex situ conservation to rescue the remaining individuals. XLS contains the greatest number of individuals and has the highest genetic diversity level; therefore, a seed bank and resource nursery should be established through breeding experiments and transplantation. Moreover, samples from the entire natural distribution range should also be collected in botanical gardens with suitable habitats to increase the chances of gene flow among populations. To summarize, a high genetic diversity level was observed in *A. miaotaiense*, but its distribution was narrow. Therefore, comprehensive protective measures should be taken along with in situ or ex situ conservation. In particular, we should attach great importance to the protection of extant *A. miaotaiense* populations and seriously implement protective strategies to maintain the high level of genetic diversity.

## Figures and Tables

**Table 1:** Summary of *A. miaotaiense* sampling locations in China.

Regions	Sampling Locality	Sample Size	Population Codes	Latitude (N)/Longitude (E)	Altitude (m)
G1	Tianshui, Gansu	149	XLS	N: 34°18.405′/E: 106°08.323′	1504
	Maiji, Gansu	17	MJ	N: 34°07.052′/E: 105°54.214′	1520
G2	Zhouzhi, Shaanxi	37	HZZ	N: 33°50.680′/E: 107°48.068′	1502
	Zhouzhi, Shaanxi	29	YDH	N: 34°50.057′/E: 107°47.395′	1500
	Foping, Shaanxi	22	BMG	N: 33°33.382′/E: 107°48.699′	1260
G3	Lantian, Shaanxi	7	TY	N: 34°02.577′/E: 107°53.075′	1293

**Table 2:** Characterization of 19 polymorphic simple sequence repeats (SSRs).

	Primer Sequences (5′ds-3′)	Repeat Motif	Ta (°C)	Expected Size (bp)	N_a_	H_o_	H_e_	PIC	HWE	NRA
BFAM-2	F:AGGAGATGGAAAGCAGGGAT R:GGGACTTTCGGACAAACAAA	(CT)_17_	60	224	10	0.449	0.777	0.752	***	1
BFAM-74	F:CTAAGACACCGTGCAAGCAA R:ACGCATGATAGGGCTCGTAA	(AT)_8_	60	196	9	0.591	0.779	0.747	***	3
BFAM-50	F:TCTCCATCTCCCCTTGGTAA R:GGAGGTTTCATGGACACGAT	(TA)_10_	60	269	8	0.397	0.800	0.772	***	2
BFAM-88	F:GAACTCCTTGTCGGATTCCA R:ATTTCCACGGACCGTACTCA	(TCT)_8_	60	243	5	0.289	0.35	0.329	***	0
BFAM-34	F:GCGGTGATGAACTGATGATG R:CACCCAAATCACCTTCGTCT	(GA)_7_	60	140	6	0.506	0.566	0.517	*	3
BFAM-109	F:GTCATCACATGCCTTTCCCT R:GCAACTCGGCTAAAGATTGC	(GAT)_8_	60	244	4	0.238	0.238	0.223	***	1
BFAM-91	F:TGACTTGTCCCTCAAATCCA R:GAAAATGGGGTGTCTCCAAA	(AT)_13_	60	206	10	0.753	0.82	0.796	***	2
BFAM-219	F:AGAGTGCAAAGCAGCAAATG R:CGTTGGGACTCATGTCAATG	(AT)_12_	60	235	8	0.813	0.841	0.820	***	1
BFAM-107	F:CAAACAGAGTCGGCTGTGAA R:TTTTGTCGGCCAGTTAAACC	(GTT)_7_	60	203	3	0.240	0.247	0.229	ns	0
BFAM-241	F:ATCAGAGAGGCGGACTTGAA R:TCATTTGCCCTCCTAATTGC	(CAA)8	60	153	8	0.708	0.735	0.699	**	2
BFAM-254	F:GGACGAATGGAGATTGGAGA R:AAAGAGCTCCAAGAGGGTGA	(AAG)_7_	59	244	3	0.076	0.153	0.147	***	0
BFAM-255	F:CGCACCTTTCAGTAATGCCT R:AGAACATGCCCACAACCTGT	(AT)_10_	60	264	13	0.882	0.822	0.801	***	2
BFAM-136	F:GTGGGGAAATGAAGGGAAAT R:GCATCTGTCCCGAATCAAAT	(TTC)_7_	60	273	4	0.455	0.448	0.372	***	0
BFAM-123	F:AGGCAGGTGTCAGTGTTTCA R:GCCAGGTGGGTTCTACAAAA	(GAA)_10_	60	208	8	0.564	0.675	0.621	***	3
BFAM-262	F:TGTTGTGTTCGTTCCATCGT R:CCTTTGGTTCCCTGACCTTT	(AT)_7_	60	243	9	0.571	0.777	0.745	***	3
BFAM-166	F:CAAATCCCGACAATCTCTCC R:TGGAGGAAGCAGTCAAGGTT	(CT)_10_	60	225	19	0.698	0.812	0.791	***	6
BFAM-263	F:TATGGGCAGTCTTGGGTTTC R:CCAGGAACAAGCATGGATTT	(TG)_10_	60	223	13	0.523	0.774	0.743	***	4
BFAM-178	F:ACCAAACCAGAGATCCAACG R:AATCTCTCACGCCCCTTTCT	(GA)_11_	60	176	5	0.667	0.675	0.631	**	1
BFAM-220	F:GCTCAACCATCCAACGATTT R:TCCAGTGGCATCAGATTGAA	(TA)_13_	60	273	7	0.620	0.775	0.740	***	1
Total					152	10.04	12.06	11.47		35
Mean					8	0.528	0.635	0.604		1.842

N_a_, number of different alleles; H_o_, observed heterozygosity; H_e_, expected heterozygosity; HWE, deviation from Hardy–Weinberg equilibrium; PIC, polymorphic information content; N_m_, number of effective migrants; NRA, number private allele; ns, not significant; * *p* < 0.05; ** *p* < 0.01; *** *p* < 0.001.

**Table 3:** Genetic diversity assessment of six populations based on 19 expressed sequence tag-derived simple sequence repeat (EST-SSRs).

	Sample Size	N_a_	N_e_	H_o_	H_e_	F	Private Alleles
XLS	149	6.053	2.965	0.512	0.573	0.088	13
MJ	17	4.263	2.783	0.586	0.561	−0.046	2
HZZ	37	5.737	3.249	0.55	0.602	0.106	9
YDH	29	4.211	2.755	0.584	0.557	−0.046	3
BMG	22	4.053	2.561	0.464	0.504	0.056	6
TY	7	4.105	3.173	0.496	0.585	0.147	2
Mean	44	4.737	2.914	0.532	0.564	0.051	5.833

**Table 4:** Nei’s genetic distance (below asterisks) and pairwise genetic differentiation index (F_st_) (above asterisks) among six populations.

	XLS	MJ	HZZ	YDH	BMG	TY
XLS	****	0.065	0.074	0.089	0.078	0.065
MJ	0.181	****	0.059	0.095	0.116	0.093
HZZ	0.251	0.218	****	0.082	0.083	0.077
YDH	0.293	0.325	0.291	****	0.103	0.091
BMG	0.227	0.401	0.267	0.313	****	0.096
TY	0.173	0.334	0.286	0.304	0.327	****

**Table 5:** Analyses of molecular variance (AMOVA) for different populations and groups of *A. miaotaiense*.

	Degrees of Freedom	Sum of Squares	Variance Components	Percentage of Variation (%)	Genetic Differentiation Index
Variance partition ^a^					
Among populations	5	633.767	3.414	18.99	F_st_ = 0.190
Within populations	255	3712.486	14.559	81.01	
Total	260	4346.253	17.973		
Variance partition ^b^					
Among groups	2	307.470	0.260	1.44	F_st_ = 0.014
Among populations within groups	3	326.298	3.215	17.83	
within populations	255	3712.486	14.559	80.73	
Total	260	4346.253	18.034		

^a^ The first Bayesian clustering analyses (BCA) included all populations as one hierarchical group. ^b^ The second BCA included three geographical groups.

## Supplementary Materials

The following are available online at http://www.mdpi.com/2073-4425/9/8/378/s1, Figure S1: Eukaryotic orthologous groups (KOG) classification of *Acer miaotaiense* unigenes, Figure S2: Distribution of the top BLASTX hits for the unigenes in the NCBI non-redundant protein (Nr) database, Figure S3: RMA regression of genetic distance and geographic distance (Km) matrix of *Acer miaotaiense* (*r*^2^ = 0.35, *p* = 0.50), Table S1: Summary of the analysis of de novo assembled of *Acer miaotaiense*, Table S2: Summary Analyses of transcriptome sequencing for *A. miaotaiense*, Table S3: The Kyoto Encyclopedia of Genes and Genomes (KEGG) annotation, Table S4: Summary of the Analyses of EST-SSRs for *A. miaotaiense*, Table S5: The number of repeats in each different motif length in the *Acer miaotaiense*, Table S6: Frequencies of different repeat motifs in SSRs in *Acer miaotaiense*, Table S7: The list of 96 SSRs with stable amplification, Table S8: The gene flow value among the six populations in *Acer miaotaiense*.
