# Supplementary material for: De Novo Transcriptome Assembly and Population Genetic Analyses for an Endangered Chinese Endemic Acer miaotaiense (Aceraceae)
Source: Genes (Basel). 2018 Jul 27;9(8):378. doi: 10.3390/genes9080378 (PMC6115825; doi:10.3390/genes9080378)
Supplement: Supplementary file 1 [file genes-09-00378-s001.zip › Supplementary/Supplementary Figures.docx]

**Supplementary Figures**


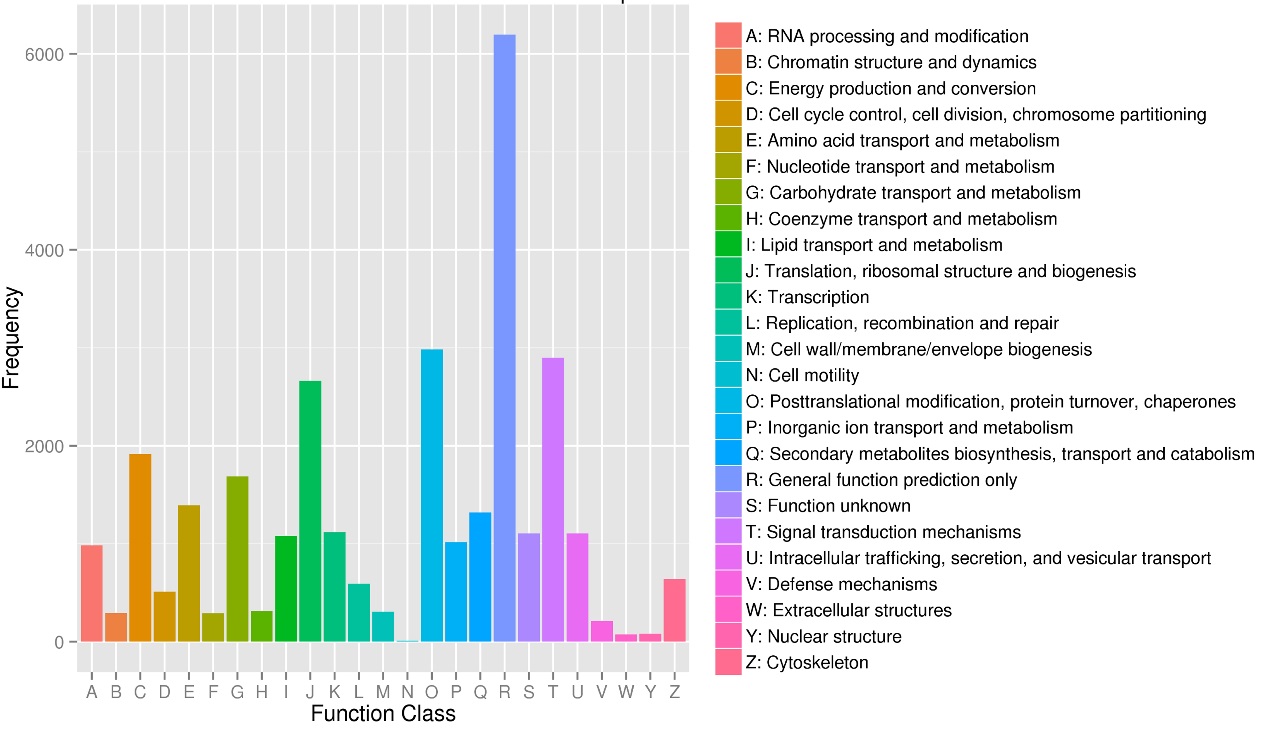


**Figure S1.** Eukaryotic orthologous groups (KOG) classification of *Acer miaotaiense* unigenes.


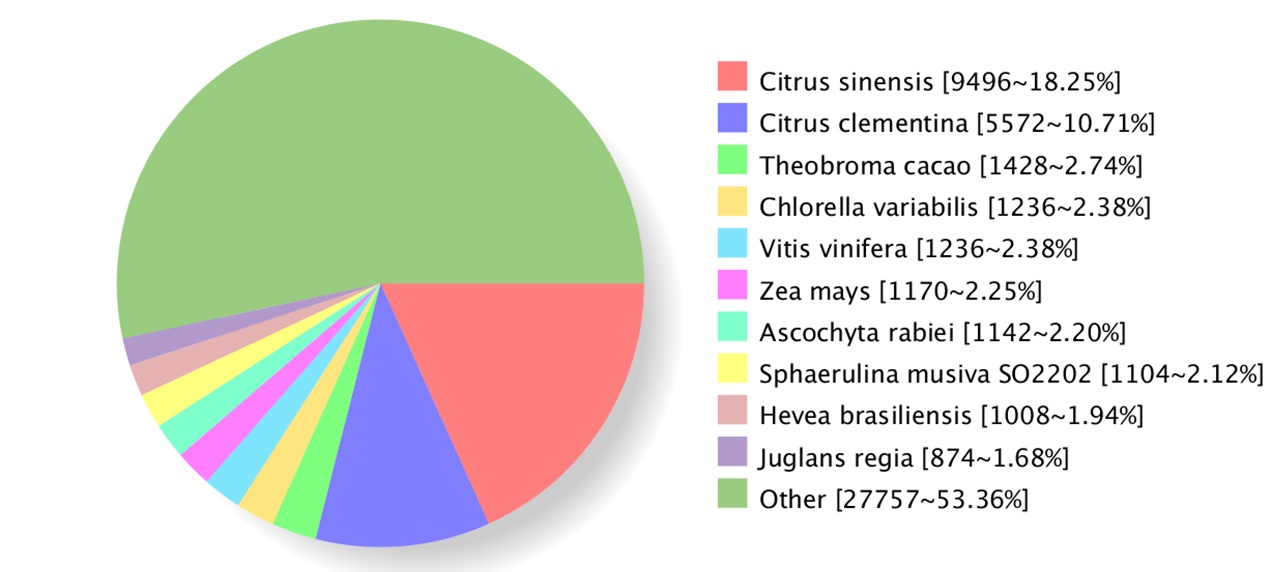


**Figure S2.** Distribution of the top BLASTX hits for the unigenes in the NCBI non-redundant protein (Nr) database.


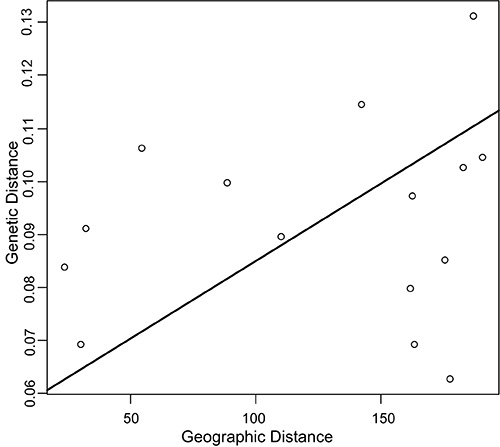


**Figure S3.** RMA regression of genetic distance and geographic distance (Km) matrix of *Acer miaotaiense.* (*r^2^* = 0.35, *p* = 0.50).
